# Sterblichkeitsentwicklung in Deutschland im internationalen Kontext

**DOI:** 10.1007/s00103-024-03867-9

**Published:** 2024-04-18

**Authors:** Pavel Grigoriev, Markus Sauerberg, Domantas Jasilionis, Alyson van Raalte, Sebastian Klüsener

**Affiliations:** 1 Bundesinstitut für Bevölkerungsforschung (BiB), Friedrich-Ebert-Allee 4, 65185 Wiesbaden, Deutschland; 2 Max-Planck-Institut für demografische Forschung, Rostock, Deutschland; 3 Demografisches Forschungszentrum, Vytautas-Magnus-Universität, Kaunas, Litauen; 4 The Max Planck – University of Helsinki Center for Social Inequalities in Population Health (MaxHel Center), Helsinki, Finnland; 5 Universität zu Köln, Köln, Deutschland

**Keywords:** Lebenserwartung, Deutschland, Internationaler Vergleich, Life expectancy, Germany, International comparison

## Abstract

**Hintergrund und Ziel:**

Deutschland hat aufgrund seiner großen Wirtschaftskraft und eines gut ausgebauten Gesundheitssystems gute Voraussetzungen für eine überdurchschnittlich starke Reduzierung der Sterblichkeit. Jedoch fällt Deutschland bei der Lebenserwartung in Westeuropa zunehmend zurück. Unsere Analyse vergleicht die Sterblichkeitsentwicklung in Deutschland mit anderen westeuropäischen Ländern zwischen 1960 und 2019. Wir untersuchen langfristige Tendenzen in der Rangposition Deutschlands im westeuropäischen Vergleich und nehmen eine detaillierte Sterblichkeitsanalyse nach Alter vor.

**Methoden:**

Die Analyse stützt sich auf Mortalitätsdaten aus der Human Mortality Database (HMD). Informationen über die Entwicklung einzelner Todesursachen stammen aus der Datenbank der World Health Organization (WHO). Für den internationalen Vergleich verwenden wir etablierte Mortalitätsindikatoren (altersstandardisierte Sterberate, Perioden-Lebenserwartung).

**Ergebnisse:**

Deutschland weist im Vergleich zu anderen westeuropäischen Ländern eine erhöhte Sterblichkeit in den mittleren und hohen Altersgruppen auf. Dabei ist Deutschlands Rückstand in der Lebenserwartung zum Durchschnitt der anderen westeuropäischen Länder gerade in den letzten 20 Jahren noch einmal angewachsen. Lag Deutschland im Jahr 2000 bei den Männern 0,73 Jahre und bei Frauen 0,74 Jahre zurück, waren es 2019 bereits 1,43 bzw. 1,34 Jahre. Dies erklärt sich überwiegend durch Sterblichkeit an nichtübertragbaren Krankheiten.

**Fazit:**

Damit Deutschland wieder zu den anderen westeuropäischen Ländern aufschließen kann, ist ein stärkerer Fokus auf eine weitere Verringerung der Sterblichkeit im Alter ab 50 Jahren erforderlich. Hierfür ist auch mehr Forschung zu den Ursachen für Deutschlands schlechtes Abschneiden notwendig.

**Zusatzmaterial online:**

Zusätzliche Informationen sind in der Online-Version dieses Artikels (10.1007/s00103-024-03867-9) enthalten.

## Abstract (en)

**Background and aim:**

Due to its strong economy and a well-developed healthcare system, Germany is well positioned to achieve above-average reductions in mortality. Nevertheless, in terms of life expectancy, Germany is increasingly falling behind Western Europe. We compare mortality trends in Germany with other Western European countries, covering the period from 1960 to 2019. The focus is on long-term trends in Germany’s ranking in international mortality trends. In addition, we conduct a detailed mortality analysis by age.

**Methods:**

Our analysis is mostly based on mortality data from the Human Mortality Database (HMD). Cause-specific mortality data originate from the database of the World Health Organization (WHO). For the international comparison of mortality trends, we use conventional mortality indicators (age-standardized mortality rate, period life expectancy).

**Results:**

Compared to other Western European countries, Germany has higher mortality in the middle and older age groups. Germany’s life expectancy gap compared to Western Europe has grown during the past 20 years. In 2000, Germany was 0.73 years behind for men and 0.74 years behind for women. By 2019, these figures had risen to 1.43 and 1.34 years, respectively. This is mainly due to mortality from non-communicable diseases.

**Conclusion:**

For Germany to catch up with other Western European countries, a stronger focus on further reducing mortality at ages 50+ is crucial. This also requires further research to understand the factors behind Germany’s disadvantageous position.

## Einleitung

Über viele Jahrzehnte wurde in den meisten ökonomisch hoch entwickelten Ländern ein stetiger Rückgang der Sterblichkeit verzeichnet [1, 2]. Vereinzelt traten jedoch in einigen Ländern auch längerfristige Abweichungen wie Stagnationsphasen oder gar Anstiege bei der Sterblichkeit auf. Zu diesen Ausnahmen zählen etwa die längerfristige Stagnationsphase bei den Frauen in Dänemark von Anfang der 1970er- bis in die Mitte der 1990er-Jahre [3], die aktuelle Mortalitätskrise in den USA [4, 5] sowie die momentan stockenden Anstiege bei der Lebenserwartung im Vereinigten Königreich [6].

Wie sich die Sterblichkeit zukünftig entwickeln wird, ist von verschiedenen Faktoren abhängig. Zu den Faktoren, die sich negativ auf die weitere Entwicklung auswirken könnten, gehört die zumindest in den letzten Jahren wieder zunehmende Bedeutung von Infektionskrankheiten (u. a. Grippewelle von 2015 und COVID-19-Pandemie). Daneben stellen auch Umwelt- und Klimaveränderungen eine potenzielle Herausforderung für eine weitere Verringerung der Sterblichkeit dar [7]. Zukünftige deutliche Verbesserungen der Lebenserwartung würden in den meisten ökonomisch hoch entwickelten Ländern auch weitere Rückgänge der Sterblichkeit in sehr hohen Altern jenseits von 80 und 90 Jahren erfordern [1]. Ob systematische Verbesserungen bei altersbedingten Erkrankungen, wie etwa Alzheimer oder chronischen Gesundheitsbeeinträchtigungen, gelingen können, ist unklar (siehe [8]). Gleichzeitig muss dem Auftreten von Mortalitätskrisen in jüngeren Altern, wie sie etwa gerade in den USA durch die *Deaths of Despair* ([4]; u. a. bedingt durch Opioidmissbrauch) verzeichnet werden, entgegengewirkt werden. Andererseits besteht aber weiterhin Potenzial für die Reduzierung von Sterblichkeit, wie etwa Studien zur vermeidbaren Mortalität aufzeigen [9]. Dies gilt in Deutschland insbesondere für Herz-Kreislauf-Erkrankungen [10, 11]. Aber auch bei Neubildungen könnte durch eine bessere Vorbeugung, Früherkennung und Behandlung noch eine erhebliche Verringerung der Sterblichkeit erreicht werden.

Im internationalen Vergleich schneidet Deutschland bereits seit längerer Zeit eindeutig unterdurchschnittlich ab. Der langjährige Rückstand in der deutschen Lebenserwartung scheint sich wesentlich durch eine höhere Sterblichkeit aufgrund von Herz-Kreislauf-Erkrankungen im fortgeschrittenen Erwachsenenalter bzw. Rentenalter zu erklären [10]. Dies verdeutlicht auch eine groß angelegte Studie über 15 Länder mit niedriger Sterblichkeit in Europa, Asien und Nordamerika. Diese Länder wurden hinsichtlich Veränderungen in der Todesursachenstruktur analysiert [12]. Zusammen mit Österreich weist Deutschland im Jahr 2015 den höchsten Anteil an Sterbefällen durch Erkrankungen des Kreislaufsystems auf. Dieser Anteil lag bei Frauen 10 Prozentpunkte über dem Durchschnittswert (43 % verglichen mit 33 %), während er für Männer 8 Prozentpunkte höher als der Durchschnitt war (38 % gegenüber 30 %). Beim prozentualen Anteil von Krebssterbefällen befand sich Deutschland dagegen unter dem Durchschnittswert der 15 Länder. Der letztgenannte Befund erklärt sich vermutlich durch sogenannte konkurrierende Risiken. Durch die hohe Sterblichkeit aufgrund von Herz-Kreislauf-Erkrankungen reduziert sich das Risiko, an Krebs zu versterben. Hierauf deuten auch Entwicklungen im Zeitverlauf hin. So ist bei allen Ländern der Studie der prozentuale Anteil von Sterbefällen durch Herz-Kreislauf-Erkrankungen über die Zeit hinweg stark gesunken, während andere Todesursachen, wie beispielsweise Krebs, anteilig zugenommen haben. Auch Deutschland befindet sich auf diesem Entwicklungspfad, liegt dabei aber etwa ein Jahrzehnt hinter den Vergleichsländern [12].

In diesem Beitrag untersuchen wir die langfristige Sterblichkeitsentwicklung in Deutschland aus einer international vergleichenden Perspektive. In einer vorhergehenden Studie haben wir aufgezeigt, dass Deutschland trotz eines hohen wirtschaftlichen Entwicklungsstands, eines stark ausgebauten Wohlfahrtsstaats und eines gut zugänglichen und leistungsfähigen Gesundheitssystems seit Langem eine verhältnismäßig niedrige Lebenserwartung aufweist [10]. Anhand dieser Studie konnten wir belegen, dass das deutsche Langlebigkeitsdefizit im Vergleich zu Vorreiterländern, wie etwa der Schweiz, Frankreich, Spanien oder Japan, vor allem auf einen Nachteil in der Sterblichkeit im höheren Erwachsenenalter zurückzuführen ist (bei Männern ab 50 Jahren, bei Frauen ab 65 Jahren). Hinsichtlich der Todesursachen erklärte sich der Rückstand insbesondere durch eine höhere Sterblichkeit aufgrund von Herz-Kreislauf-Erkrankungen. Dies gilt selbst im Vergleich zu anderen „Nachzüglerländern“ wie den USA und dem Vereinigten Königreich. Die immer noch hohe kardiovaskuläre Sterblichkeit in Deutschland scheint auch auf unzureichende Prävention und Primärversorgung zurückzuführen zu sein (siehe [10]).

Der vorliegende Beitrag erweitert die Diskussion über den deutschen Lebenserwartungsrückstand im Verhältnis zu Ländern mit ähnlichem ökonomischen Entwicklungsstand in mehrfacher Hinsicht. Erstens vergleichen wir die Sterblichkeitsentwicklung in Deutschland mit einer größeren Anzahl von Ländern. Dabei konzentrieren wir uns auf die Mitglieder der Europäischen Union vor der ersten Osterweiterung im Jahr 2004 (EU–15), welche überwiegend in Westeuropa liegen, sowie auf die Schweiz. Zweitens untersuchen wir die langfristigen Trends und Muster von Deutschlands Positionierung in internationalen Rangfolgen bei der Sterblichkeit. Drittens präsentieren wir eine detaillierte Analyse der Muster nach Alter. Der Schwerpunkt unserer Analyse liegt auf dem Zeitraum seit der deutschen Wiedervereinigung im Jahr 1990. Um die seit 1990 beobachteten Muster in einen längerfristigen Kontext setzen zu können, ergänzen wir unsere Analyse mit einer Auswertung grundlegender Mortalitätsindikatoren seit 1960.

## Theoretischer Hintergrund

Nachdem die Lebenserwartung in den 1960er-Jahren in vielen Ländern stagnierte, stieg sie ab den 1970er-Jahren in vielen westlichen Ländern mit hohem ökonomischen Entwicklungsstand wieder an. Hierzu trugen insbesondere Fortschritte bei der Reduzierung von Herz-Kreislauf-Erkrankungen bei. Diese zweite (moderne) Phase des gesundheitlichen Übergangs [13, 14], welche sich sowohl durch grundlegende Veränderungen bei den Risikofaktoren im Verhalten als auch durch Fortschritte in der Medizintechnik und der Krankheitsprävention erklärt, wird in der Literatur auch als „kardiovaskuläre Revolution“ bezeichnet [1, 15].

Um die Mechanismen zu erklären, die systematische Muster beim Auseinanderdriften und Angleichen von Sterblichkeitsniveaus zwischen Ländern bzw. Gruppen von Ländern bedingen, haben France Meslé und Jacques Vallin die Theorie der Konvergenz-Divergenz-Zyklen bei der Sterblichkeit entwickelt [1]. Im Zentrum dieser Theorie stehen bedeutende medizinische Innovationen, welche die Überlebenschancen erheblich erhöhen. Hierzu zählen etwa die Entwicklung von Antibiotika oder von Medikamenten zur Senkung von Bluthochdruck. Derartige Innovationen erhöhen laut dieser Theorie in einer ersten Phase die Ungleichheit zwischen Ländern. Dies hängt damit zusammen, dass es in der Regel einigen Vorreiterländern im Vergleich zu anderen Ländern besser gelingt, die Innovationen schnell breiten Bevölkerungsschichten verfügbar zu machen. Dazu tragen Unterschiede im wirtschaftlichen, sozialen und politischen Umfeld bei. In einer zweiten Phase können aber in der Regel auch die Bevölkerungen in den zunächst zurückfallenden Ländern von der medizinischen Innovation profitieren, was dann wieder Konvergenztendenzen befördert. Insofern kann es immer wieder aufgrund von großen Durchbrüchen beim medizinischen Fortschritt zu Konvergenz-Divergenz-Zyklen kommen [1].

Unterschiede zwischen den Ländern spiegeln nicht nur Disparitäten bei den verfügbaren Ressourcen wider, sondern auch Differenzen in der Gesundheitspolitik – etwa hinsichtlich der Prävention, Früherkennung und Behandlung von Erkrankungen. Empirische Belege deuten darauf hin, dass nationale gesundheitspolitische Maßnahmen etwa bei der Eindämmung von Tabak- und Alkoholkonsum, der Vorbeugung und Behandlung von Bluthochdruck, der Krebsvorsorge, der Straßenverkehrssicherheit, bei Lebensmittelstandards und Ernährung, Kindergesundheit sowie Infektionskrankheiten und Luftverschmutzung in den letzten vier Jahrzehnten in vielen europäischen Ländern zu erheblichen Verbesserungen der Gesundheit der Bevölkerung beigetragen haben [16]. Die Tatsache, dass benachbarte Länder mit ähnlichen sozioökonomischen Bedingungen mitunter sehr unterschiedliche Gesundheitsergebnisse aufweisen, deutet darauf hin, dass sich Länder im Grad der Umsetzung gesundheitspolitischer Maßnahmen zum Teil erheblich unterscheiden [16–18].

## Methoden

Unsere Analyse stützt sich hauptsächlich auf Mortalitätsdaten aus der Human Mortality Database (HMD; [19]) für den Zeitraum ab 1960. Die Datenbank enthält Sterbefälle und Bevölkerungszahlen aufgegliedert nach einzelnen Altersjahren. Wir konzentrieren uns auf die vergleichende Analyse langfristiger Sterblichkeitstrends und Muster der Sterblichkeit nach Alter zwischen Deutschland und den alten Mitgliedstaaten der Europäischen Union (EU–15) sowie der Schweiz. Dabei endet unser Untersuchungszeitraum vor der COVID-19-Pandemie, also im Jahr 2019. Wir schließen Griechenland von unserer Analyse aus, da belastbare Sterblichkeitsdaten für dieses Land erst ab 1981 vorliegen. Folglich vergleichen wir Deutschland mit 14 Ländern: Darunter sind 13 weitere Länder der EU–15 (Belgien, Dänemark, Finnland, Frankreich, Irland, Italien, Luxemburg, die Niederlande, Österreich, Portugal, Schweden, Spanien, Vereinigtes Königreich) sowie die Schweiz. Der Einfachheit halber bezeichnen wir diese Gruppe von 14 Ländern als „Westeuropa“. Während altersspezifische Sterblichkeitsraten und andere Sterblichkeitsindikatoren für die einzelnen Länder leicht verfügbar sind, mussten wir diese für die zusammengefasste Westeuropa-Gruppe berechnen. Dafür wurden Sterbefälle und Bevölkerungszahlen aggregiert, um Sterblichkeitsraten und mithilfe von klassischen Sterbetafeln die Perioden-Lebenserwartung zu ermitteln [20].

Um in einer Dekompositionsanalyse die Altersgruppen zu bestimmen, die für den Unterschied in der Lebenserwartung zwischen Deutschland und der Westeuropa-Gruppe verantwortlich sind, haben wir den sogenannten *Stepwise-Replacement-*Algorithmus verwendet [21]. Dieser Algorithmus ist in dem R‑Paket *DemoDecomp* [22] enthalten und ermöglicht die Zerlegung der Differenz zwischen zwei Lebenserwartungen in die Beiträge einzelner Altersgruppen. Eine formale Beschreibung des Algorithmus findet sich bei Andreev et al. 2002 [21], während van Raalte und Nepomuceno [23] die Methode grafisch veranschaulicht haben. Der *Stepwise-Replacement-*Algorithmus dient dazu, durch das Austauschen einzelner Sterberaten und einer anschließenden Neuberechnung der Lebenserwartung den Einfluss jeder altersspezifischen Sterberate auf die Differenz zweier Lebenserwartungswerte zu ermitteln.

Außerdem vergleichen wir die altersspezifischen Sterberaten einzelner Länder, indem wir sie mathematisch ins Verhältnis zueinander setzen. Die dabei berechneten *Mortality Rate Ratios* (MRR) werden anschließend mithilfe von Lexis-Oberflächen visualisiert. Um zufällige Schwankungen durch kleine Sterbefallzahlen zu minimieren, haben wir vor der Berechnung der MRR zunächst die beobachteten altersspezifischen Sterberaten mit dem R‑Paket *MortalitySmooth* [24] geglättet. Die geglätteten Sterberaten können aussagekräftiger visualisiert werden, da die Lexis-Oberflächen dann weniger Rauschen durch jährliche Zufallsfluktuationen enthalten. Hierdurch können reale Sterblichkeitstrends besser identifiziert werden.

Eine weitere vergleichende Analyse basiert auf der Rangfolge der Länder bei der Lebenserwartung in einem bestimmten Alter. Wir ergänzen diese Analyse, indem wir die Position Deutschlands im Vergleich zu einzelnen westeuropäischen Ländern mit Bezug auf die Sterblichkeit nach großen Gruppen von Todesursachen einordnen. Um die Todesfälle nach bestimmten Todesursachen unabhängig von der Altersstruktur des jeweiligen Landes vergleichen zu können, verwenden wir die altersstandardisierten Sterberaten aus der Datenbank der Weltgesundheitsorganisation (WHO; [25]). Diese basieren auf der Altersstruktur der WHO-Weltbevölkerung.

## Ergebnisse

**Figure 1:**
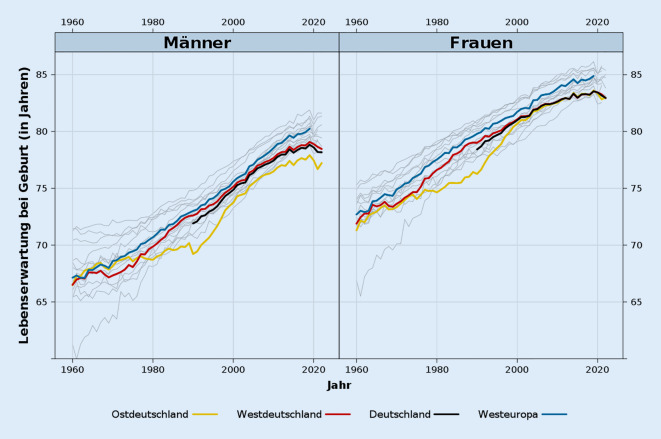

Abb. 1 veranschaulicht die zeitliche Entwicklung der Lebenserwartung in Deutschland in den letzten sechs Jahrzehnten im Vergleich zum Durchschnitt der Westeuropa-Länder ohne Deutschland (blaue Linie). Zusätzlich werden Trends für Ost- und Westdeutschland gezeigt. Die grauen Linien stellen den Verlauf in den einzelnen Westeuropa-Ländern dar. In den frühen 1960er-Jahren lag die Lebenserwartung sowohl in Ost- als auch in Westdeutschland in der Nähe des durchschnittlichen Niveaus der Westeuropa-Länder. Erste nennenswerte Abweichungen traten Ende der 1960er-Jahre auf. Damals kam es im Kontext der Hongkong-Grippeepidemie im Winter 1968/1969 in praktisch allen Ländern zu einem erheblichen Rückgang der Lebenserwartung, wobei westdeutsche Männer besonders betroffen waren. Im Gegensatz zu Westdeutschland verlief die Entwicklung der männlichen Lebenserwartung in Ostdeutschland bis Anfang der 1970er-Jahre parallel zum Westeuropa-Niveau und stagnierte erst danach.

Ein wichtiger Wendepunkt in der Sterblichkeitsentwicklung in Deutschland erfolgte Mitte der 1970er-Jahre. Zu diesem Zeitpunkt begann Westdeutschland im Kontext der kardiovaskulären Revolution den Rückstand zum westeuropäischen Durchschnitt zu reduzieren. Diese Konvergenzphase war vor allem bei den Männern ausgeprägt, die Mitte der 1980er-Jahre den Abstand zu Westeuropa fast aufholen konnten. Im Gegensatz dazu war die Sterblichkeitsentwicklung in Ostdeutschland eher durch Stagnation oder nur leichte Verbesserungen gekennzeichnet. Infolgedessen erreichte der Sterblichkeitsabstand zwischen den beiden Teilen Deutschlands zum Zeitpunkt der Wiedervereinigung im Jahr 1990 den Höchststand innerhalb des beobachteten Zeitraums. Dank massiver finanzieller Investitionen in die Gesundheitsversorgung und das Wohlergehen der ostdeutschen Bevölkerung verringerte sich dieser Unterschied in den 1990er-Jahren jedoch sehr schnell [26]. Bis Anfang der 2000er-Jahre hatte die Lebenserwartung der Frauen in Ostdeutschland zu Westdeutschland aufgeschlossen und auch gegenüber dem restlichen Westeuropa erheblich aufgeholt. Die ostdeutschen Männer konnten zunächst ebenfalls den Abstand gegenüber Westdeutschland und dem restlichen Westeuropa reduzieren. In den letzten zwei Jahrzehnten verharrte deren Lebenserwartung aber gegenüber Westdeutschland bei einem Rückstand von einem Jahr.

**Figure 2:**
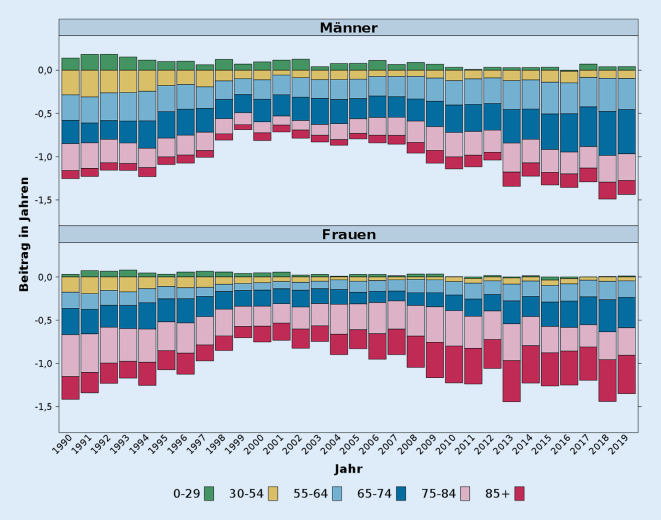

Der Beginn der 2000er-Jahre markiert einen weiteren Wendepunkt in der Dynamik der Sterblichkeitsentwicklung in Deutschland. Seitdem hat die Sterblichkeitslücke zwischen Deutschland und den Westeuropa-Ländern stetig zugenommen. Während sie im Jahr 2000 bei Männern 0,73 Jahre und bei Frauen 0,74 Jahre betrug, lag sie 2019 (dem letzten Jahr vor der Pandemie) bereits bei 1,43 bzw 1,34 Jahren. In Abb. 2 wird dieses Zurückfallen hinter dem restlichen Westeuropa genauer unter die Lupe genommen. Anhand einer Dekomposition wird für den Zeitraum ab 1990 aufgezeigt, welche Alter zu der sich öffnenden Lebenserwartungslücke zwischen Deutschland und dem restlichen Westeuropa besonders stark beigetragen haben. Abgesehen von der Bevölkerung unter 30 Jahren hatten Männer und Frauen in Deutschland seit der Wiedervereinigung eine höhere Sterblichkeit als Männer und Frauen in den Westeuropa-Ländern. Die größten Unterschiede bestehen dabei in der Bevölkerung ab 65 Jahren, wobei diese bei Frauen besonders ausgeprägt sind. Bei diesen trägt insbesondere die Sterblichkeit im Alter von 75 und mehr Jahren zu der Lücke bei. Aktuell erklärt bei Frauen allein die Altersgruppe ab 85 Jahren etwa ein Viertel des Unterschieds zu den Westeuropa-Ländern. Im Gegensatz dazu wird bei den Männern das Zurückfallen von Deutschland bei der Sterblichkeit weitgehend von den Altersgruppen 65–74 Jahre und auch 55–64 Jahre getragen. Die Sterblichkeit im Alter von 75 und mehr Jahren spielt bei Männern aber ebenfalls eine Rolle, wobei deren Bedeutung für den Rückstand über die Zeit zugenommen hat.

Bei den konvergierenden Trends in den 1990er- und frühen 2000er-Jahren liegt die Vermutung nahe, dass diese Muster auf den raschen Rückgang der Sterblichkeitsraten in Ostdeutschland im Zuge der deutschen Wiedervereinigung zurückzuführen sind. Um dies zu überprüfen, haben wir eine Grafik analog zu Abb. 2 nur für Westdeutschland erstellt (Abb. A1 im Onlinematerial). Diese zeigt auf, dass die Trendmuster jenen in Abb. 2 sehr ähnlich sind. Insofern erklärt sich die Reduzierung der Sterblichkeitslücke zu Westeuropa in dieser Zeit nicht nur mit der Entwicklung in Ostdeutschland.

**Figure 3:**
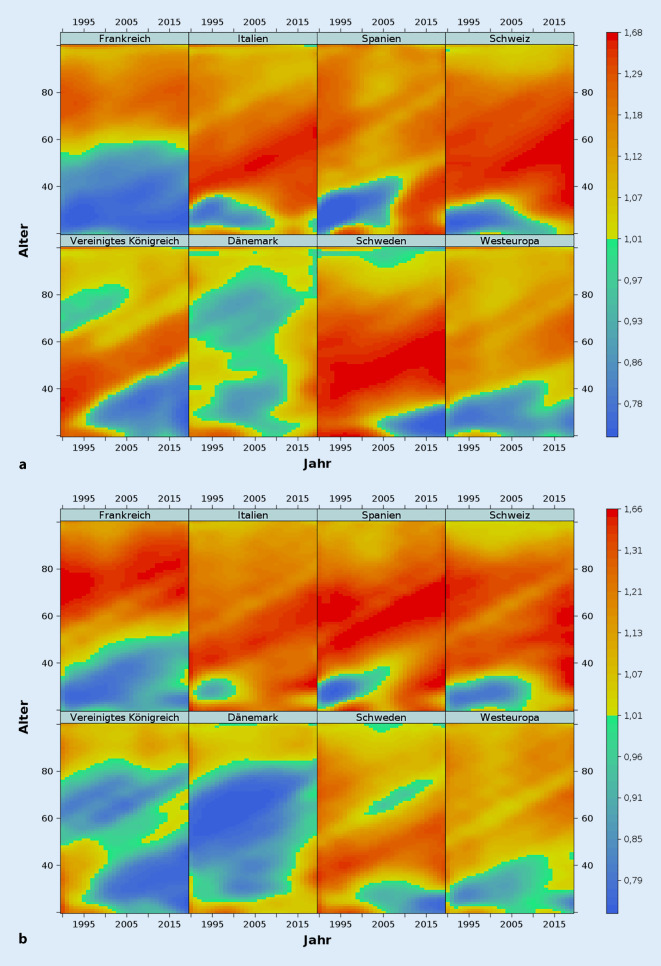

Da die Westeuropa-Länder in Bezug auf die Altersstruktur der Sterblichkeit eine recht heterogene Gruppe darstellen, werden im Folgenden Vergleiche zwischen Deutschland und sieben ausgewählten Einzelländern vorgenommen. Frankreich, Italien, Spanien und das Vereinigte Königreich wurden wegen ihrer großen Bevölkerungen bei gleichzeitig unterschiedlichen Sterblichkeitsprofilen ausgewählt. Die Schweiz und Dänemark repräsentieren Nachbarländer Deutschlands mit einer hohen bzw. niedrigen Lebenserwartung im internationalen Vergleich. Zusätzlich wird Schweden als größter Vertreter der nordischen Länder betrachtet. In den Abb. 3a (Männer) und b (Frauen) zeigen wir Lexis-Oberflächen mit den MMR. Alter unter 20 Jahren und über 100 Jahren sind ausgelassen, da die Sterblichkeitsraten sehr gering (oder gleich Null) und die Sterblichkeitsquotienten daher nicht stabil sind. Jedes kleine Quadrat stellt ein einfaches Verhältnis zwischen der in einem bestimmten Kalenderjahr in Deutschland beobachteten altersspezifischen Sterblichkeitsrate und der Sterblichkeitsrate in einem der Vergleichsländer dar. Ein Verhältnis über 1 bedeutet, dass die verzeichnete Sterblichkeit in Deutschland in diesem Alter und Jahr höher ist als im Vergleichsland, während sie bei einem Wert unter 1 niedriger ist.

Bei Männern ist die Sterblichkeit in Deutschland im Vergleich zu Frankreich in den letzten drei Jahrzehnten konstant in den Altersgruppen unter 55 Jahren niedriger und in den Altersgruppen über 60 Jahren höher gewesen. Während Männer in Deutschland in den 1990er- und frühen 2000er-Jahren einen gewissen Vorteil gegenüber Italien, Spanien und der Schweiz hinsichtlich einer niedrigeren Sterblichkeit in einigen Altersgruppen unter 40 Jahren hatten, ist dieser Vorteil in den letzten Jahren verschwunden. Es gibt praktisch kein Alter mehr, in dem Deutschland vor diesen drei Ländern platziert ist. Der Vergleich der Sterblichkeitsraten mit dem Vereinigten Königreich zeigt dagegen ganz andere Muster. Hier haben Männer in Deutschland in jungen Jahren tendenziell eine niedrigere Sterblichkeit. Der Nachteil des Vereinigten Königreichs in der Altersgruppe der unter 50-Jährigen hat in den letzten Jahren tendenziell zugenommen. Gegenüber Dänemark hatte Deutschland in der Vergangenheit bei der Überlebensrate von Männern einen leichten Vorteil. Dieser ist jedoch in den letzten Jahren verschwunden. Die vergleichende Analyse der Altersmuster der Sterblichkeit bei Frauen (Abb. 3b) zeigt einige Unterschiede zu den Männern. So ist der Vorteil Deutschlands gegenüber Frankreich in Bezug auf die niedrigere Sterblichkeit in jüngeren Jahren weniger ausgeprägt als bei den Männern. Außerdem ist die nachteilige Position Deutschlands im Vergleich zu Spanien bei den Frauen noch deutlicher.

Sowohl bei Männern als auch bei Frauen scheint es beim Vergleich zwischen Deutschland und dem Vereinigten Königreich ausgeprägte Kohortenmuster zu geben, die wahrscheinlich durch das Rauchen bedingt sind [6]. Dieser Befund sollte jedoch mit Vorsicht interpretiert werden, da die vorliegende Analyse auf Periodendaten und nicht auf Kohortendaten basiert [27]. Des Weiteren war der in der Vergangenheit in vielen Altersgruppen verzeichnete Vorsprung Deutschlands gegenüber Dänemark bei den Frauen stärker ausgeprägt als bei den Männern. Die schlechte Position der Frauen in Dänemark erklärt sich durch die starken Auswirkungen der „Rauchpandemie“ im nördlichen Nachbarland [3]. Die Rauchpandemie breitete sich in Deutschland unter Frauen zum Teil später aus, wodurch ihre Auswirkungen auf die Sterblichkeit erst jetzt ihren Höhepunkt erreichen [28]. Durch diesen zeitlichen Versatz ist zukünftig mit einer weiteren Verschlechterung der relativen Position der deutschen Frauen nicht nur im Vergleich zu Dänemark, sondern auch zu anderen Ländern zu rechnen.

**Figure 4:**
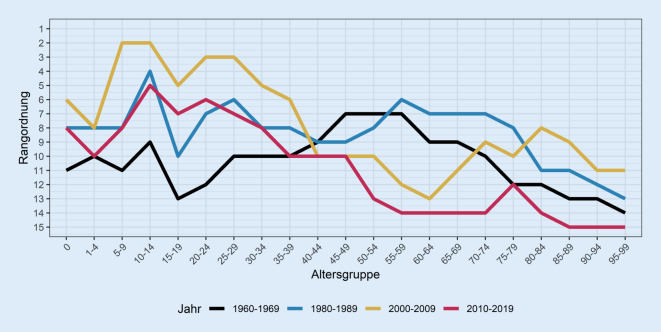

Wir wenden uns nun der Positionsentwicklung Deutschlands bei der Sterblichkeit innerhalb der 15 betrachteten westeuropäischen Länder zu. Abb. 4 zeigt die Position Deutschlands entlang der beiden Dimensionen Alter und Zeit. Bis 1990 werden dafür die Werte für Westdeutschland genutzt, ab 1990 für Gesamtdeutschland. Aus Gründen der Übersichtlichkeit werden die 1970er- und 1990er-Jahre nicht dargestellt. Eine hohe Rangposition entspricht einer vergleichsweise geringen Sterblichkeit in der jeweiligen Altersgruppe, eine niedrige Rangposition einer hohen Sterblichkeit. Wir sehen beispielsweise für die Altersgruppen 5–9 sowie 10–14 Jahre im Zeitraum 2000 bis 2009, dass lediglich ein anderes Land der 14 Vergleichsländer eine niedrigere Sterblichkeit als Deutschland aufweist. Dementsprechend befindet sich Deutschland hier auf Rang 2.

Bereits in den 1960er-Jahren (schwarze Linie) schnitt Westdeutschland bei den Sterberaten im Ländervergleich eher schlecht ab. In den jungen und mittleren Altersgruppen schwankte Westdeutschland damals im Mittelfeld zwischen dem 7. und 13. Rang. Zu den höheren Altern hin war die Position zunehmend schlechter. Lag Westdeutschland damals beim Alter 55–59 Jahre noch auf Rang 7, so war es ab 75 Jahren unter den letzten vier Ländern und bei der höchsten betrachteten Altersgruppe 95–99 Jahre an vorletzter Position. Im Zeitraum 1980–1989 schnitt Westdeutschland in der Rangliste etwas besser ab. Damals dominierten bei den meisten Altern mittlere Ränge. Bei Altersgruppen über 80 Jahren war Westdeutschland aber auch damals eher auf den hinteren Rängen. Anfang der 2000er-Jahre lag Deutschland bei den jungen Altersgruppen überwiegend auf vorderen Plätzen. Ab dem Alter 40 Jahre schnitt es jedoch deutlich schlechter ab und nahm mittlere bis hintere Ränge ein. In der letzten beobachteten Periode 2010–2019 ist Deutschland bereits in der Altersgruppe 35–39 auf einem relativ niedrigen 10. Platz. Im höheren Alter büßte Deutschland im Vergleich zu den 2000er-Jahren deutlich an Positionen ein und verzeichnet bei Altern ab 50 Jahren überwiegend letzte und vorletzte Plätze. Die letzten Plätze konzentrieren sich dabei auf die Alter über 85 Jahren.

**Figure 5:**
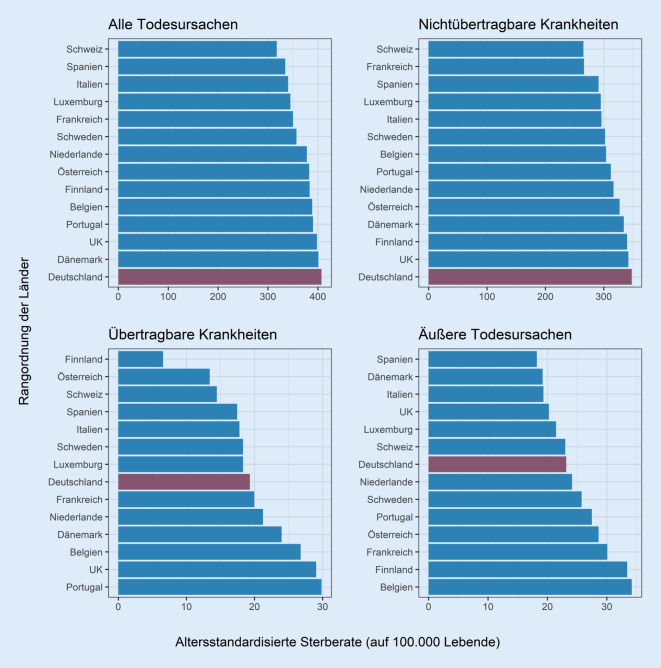

Abschließend zeigt Abb. 5 für das Jahr 2017 für alle Länder die altersstandardisierten Sterberaten für die allgemeine und die todesursachenspezifische Sterblichkeit. Sowohl bei der Gesamtsterblichkeit als auch bei den Todesfällen durch nichtübertragbare Krankheiten befindet sich Deutschland auf dem letzten Platz. Dies ist auf eine vergleichsweise hohe Sterblichkeit durch Herz-Kreislauf-Erkrankungen zurückzuführen (Abb. A2–A5 im Onlinematerial). Da Herz-Kreislauf-Erkrankungen in den meisten europäischen Ländern zu den häufigsten Todesursachen zählen, fallen diese Todesfälle überdurchschnittlich stark ins Gewicht. Hierdurch tragen diese besonders zu Deutschlands schlechtem Abschneiden im westeuropäischen Ländervergleich bei. Die Sterblichkeit durch die zweithäufigste Todesursache in Deutschland, bösartige Neubildungen, liegt im Vergleich zu den ausgewählten Ländern ebenfalls über dem Durchschnitt. Bei den weniger verbreiteten äußeren Todesursachen (z. B. Unfälle oder Suizide) oder bei Todesfällen durch übertragbare Krankheiten befindet sich Deutschland hingegen auf einem mittleren Platz.

## Diskussion

In diesem Beitrag haben wir gezeigt, dass die Lebenserwartung in Deutschland nicht nur seit Jahrzehnten hinter der im restlichen Westeuropa zurückbleibt, sondern dass sich der Rückstand seit den 2000er-Jahren vergrößert hat. Um diese zurückfallende Entwicklung umzukehren, müsste die Sterblichkeit in Deutschland schneller gesenkt werden als in anderen Ländern, was kurzfristig wahrscheinlich nur schwer zu erreichen ist.

Um Deutschlands Rückstand bei der Lebenserwartung in Westeuropa zu überwinden, müsste insbesondere in höheren Altern eine weitere Verringerung der Sterblichkeit erzielt werden. Diesbezüglich scheint gerade bei Herz-Kreislauf-Erkrankungen Handlungsbedarf zu bestehen. Die genauen Gründe für den Widerspruch zwischen einer gut finanzierten, technologisch fortschrittlichen und gut zugänglichen Gesundheitsversorgung und der schlechten Platzierung Deutschlands bei der Lebenserwartung insbesondere im Bereich der Sterblichkeit durch Herz-Kreislauf-Erkrankungen sind noch nicht ausreichend erforscht [10]. Repräsentative und international vergleichbare Daten zur Prävalenz von Herz-Kreislauf-Erkrankungen und deren Risikofaktoren sind immer noch sehr rar. Wie bei Jasilionis et al. [10] beschrieben, gibt es keine überzeugenden und systematischen Belege dafür, dass Deutschland bei der Prävalenz vieler bekannter Risikofaktoren, wie beispielsweise Rauchen, Übergewicht und körperlicher Aktivität, deutlich schlechter abschneidet als andere Länder mit einem ähnlich hohen Wohlstandsniveau. Allerdings weisen internationale Daten darauf hin, dass die Bevölkerung in Deutschland durchschnittlich schlechtere Ernährungsgewohnheiten aufweist. Dies gilt etwa für das geringere Angebot an Gemüse und Obst und dessen vergleichsweise mäßigen Konsum [29]. Auch wenn das Rauchen aktuell wenig Einfluss auf die Lücke zum restlichen Westeuropa hat, ist zu erwarten, dass die rauchbedingte Sterblichkeit bei Frauen in Deutschland in den kommenden Jahrzehnten im westeuropäischen Vergleich überdurchschnittlich zunehmen wird [30]. Diesbezüglich ist wichtig zu erwähnen, dass Deutschland unter den ökonomisch hoch entwickelten Ländern über einen langen Zeitraum hinweg im internationalen Vergleich einen der letzten Plätze in Bezug auf die öffentliche Gesundheitspolitik einnahm. Dies gilt insbesondere in den Bereichen Tabak- und Alkoholprävention sowie Ernährung [31, 32].

In vielen Fällen sind Todesursacheninformationen erforderlich, um klarere Erkenntnisse über den Einfluss von verschiedenen Risikofaktoren und von der Gesundheitspolitik auf die aktuelle und zukünftige Sterblichkeit zu gewinnen. Auch wenn die internationale Vergleichbarkeit von todesursachenspezifischen Daten teils in der Kritik steht [33], sollte die Verwendung von großen Todesursachsachengruppen wie etwa der Gruppe der Herz-Kreislauf-Erkrankungen für die meisten Altersgruppen belastbare Ergebnisse erzielen [34]. Es wäre aber eine Analyse der Mortalität nach detaillierteren Ursachen innerhalb der ICD (*International Statistical Classification of Diseases and Related Health Problems*)-Kapitel erforderlich, um den Einfluss verschiedener Risikofaktoren besser verstehen zu können. Diesbezüglich stellt es eine Herausforderung dar, dass in Deutschland Datenlimitationen die internationale Vergleichbarkeit einschränken. Diese Limitationen sind u. a. durch die dezentrale Datenerhebung und Kodierung sowie eine nur langsam erfolgende Implementierung von Kodierungssoftware (IRIS/MUSE) in den einzelnen Bundesländern bedingt [35]. Neben einer größeren Harmonisierung des Prozesses der Datenerhebung und -kodierung eröffnet die statistische Erfassung multikausaler Mortalitätsdaten im Kontext von ICD-11 neue Möglichkeiten für eine tiefgreifende Analyse der Sterblichkeit. Auch bei dieser Umstellung liegt Deutschland leider hinter vielen europäischen Ländern zurück.

Die auffallend hohe Morbidität durch Herz-Kreislauf-Erkrankungen, komplexe chirurgische Behandlungen und übermäßige Krankenhausaufenthaltsraten sowie der hohe Prozentsatz von Patientinnen und Patienten mit Herz-Kreislauf-Erkrankungen, welche Krankenhäuser erst in fortgeschrittenen Krankheitsstadien und mit Multimorbidität erreichen, deuten auf Versäumnisse bei der Prävention, Früherkennung und Behandlung hin [36]. Eine unzureichende Früherkennung und ein geringes Bewusstsein wurden auch bei anderen Krankheiten beobachtet, die ein erhöhtes Risiko für kardiovaskuläre Ereignisse aufweisen. Dies gilt etwa für chronische Nierenerkrankungen [37]. Es sind jedoch eingehendere und landesweit repräsentative Studien erforderlich, um den Einfluss dieser Herausforderungen auf die Gesundheit der Bevölkerung in Deutschland genauer zu bestimmen. Die NAKO-Gesundheitsstudie, für die nun erste Daten verfügbar sind, bietet hier großes Potenzial [38].

## Fazit

Obwohl Deutschland im internationalen Vergleich sehr viele Ressourcen in sein Gesundheitssystem investiert [10], fällt es bei der Lebenserwartung zunehmend im westeuropäischen Vergleich zurück. Gleichzeitig stehen Deutschland und das Gesundheitssystem durch die Alterung der Bevölkerung in den nächsten Jahrzehnten vor großen Herausforderungen. Allein schon der vorhergesagte Anstieg der Bevölkerung im Alter über 80 Jahren von aktuell 6 auf etwa 9 Mio. Ende der 2040er-Jahre [39] deutet darauf hin, dass bei einer Beibehaltung des Status quo hinsichtlich der Gesundheit der Bevölkerung der Bedarf an Gesundheitsleistungen noch einmal deutlich steigen würde. Dies würde die im Vergleich zu anderen ökonomisch hoch entwickelten Ländern ohnehin überhöhten Gesundheitsausgaben noch weiter in die Höhe treiben. Um die Nachhaltigkeit der Finanzierung und des Funktionierens des Gesundheitswesens zu gewährleisten, erscheint eine Diskussion über eine Neuadjustierung von Prioritäten und Investitionen im Gesundheitswesen dringend angebracht. Diesbezüglich scheint eine stärkere Fokussierung auf die Prävention und Früherkennung chronischer Krankheiten viel Potenzial zu bieten [11]. Diese Verlagerung sollte zeitnah erfolgen, damit auch die stark besetzten Babyboomer-Kohorten noch davon profitieren und gesünder altern können. Angesichts der hohen Bedeutung von Lebensstilen für Sterblichkeitsunterschiede könnte ein weiteres Zurückdrängen des Konsums von Tabakwaren, Alkohol oder anderen Suchtmitteln ebenfalls zu weiteren Fortschritten beitragen. Mehr Forschungsarbeiten und umfassendere Daten sind erforderlich, um die Rolle anderer kontextbezogener nichtmedizinischer Faktoren besser einschätzen zu können. Dies umfasst etwa formelle und informelle soziale und familiäre Netzwerke, Wertorientierungen und soziokulturelle Merkmale. So hat sich etwa die Lebenserwartung ostdeutscher Frauen dem westdeutschen Niveau angenähert. Bei den ostdeutschen Männern hingegen verbleibt bei der Lebenserwartung nach wie vor ein Rückstand von einem Jahr. Künftige Studien sollten sich auf die fortbestehenden oder sogar zunehmenden Sterblichkeitsunterschiede jenseits von Ost-West-Disparitäten konzentrieren. Dies gilt etwa für kleinräumige Unterschiede und für die erheblichen Sterblichkeitsunterschiede zwischen sozioökonomischen Gruppen. Diesbezüglich stellt es für die Forschung auch eine Herausforderung dar, dass sie in Deutschland beim Zugang zu aussagekräftigen Daten mit besonders stark ausgeprägten Limitationen konfrontiert ist.

### Supplementary Information




