# Supplementary material for: Mortality trends in Germany in an international context
Source: Bundesgesundheitsblatt Gesundheitsforschung Gesundheitsschutz. 2024 Apr 18;67(5):493–503. [Article in German] doi: 10.1007/s00103-024-03867-9 (PMC11093834; doi:10.1007/s00103-024-03867-9)
Supplement: Supplementary file 1 [file 103_2024_3867_MOESM1_ESM.pdf]

Onlinematerial zum Beitrag

„Sterblichkeitsentwicklung in Deutschland im internationalen Kontext“

Abb. A1. Beiträge von Altersgruppen zum Unterschied in der Lebenserwartung bei Geburt; Westdeutschland versus Westeuropa-Länder (ohne Deutschland), 1990–2019

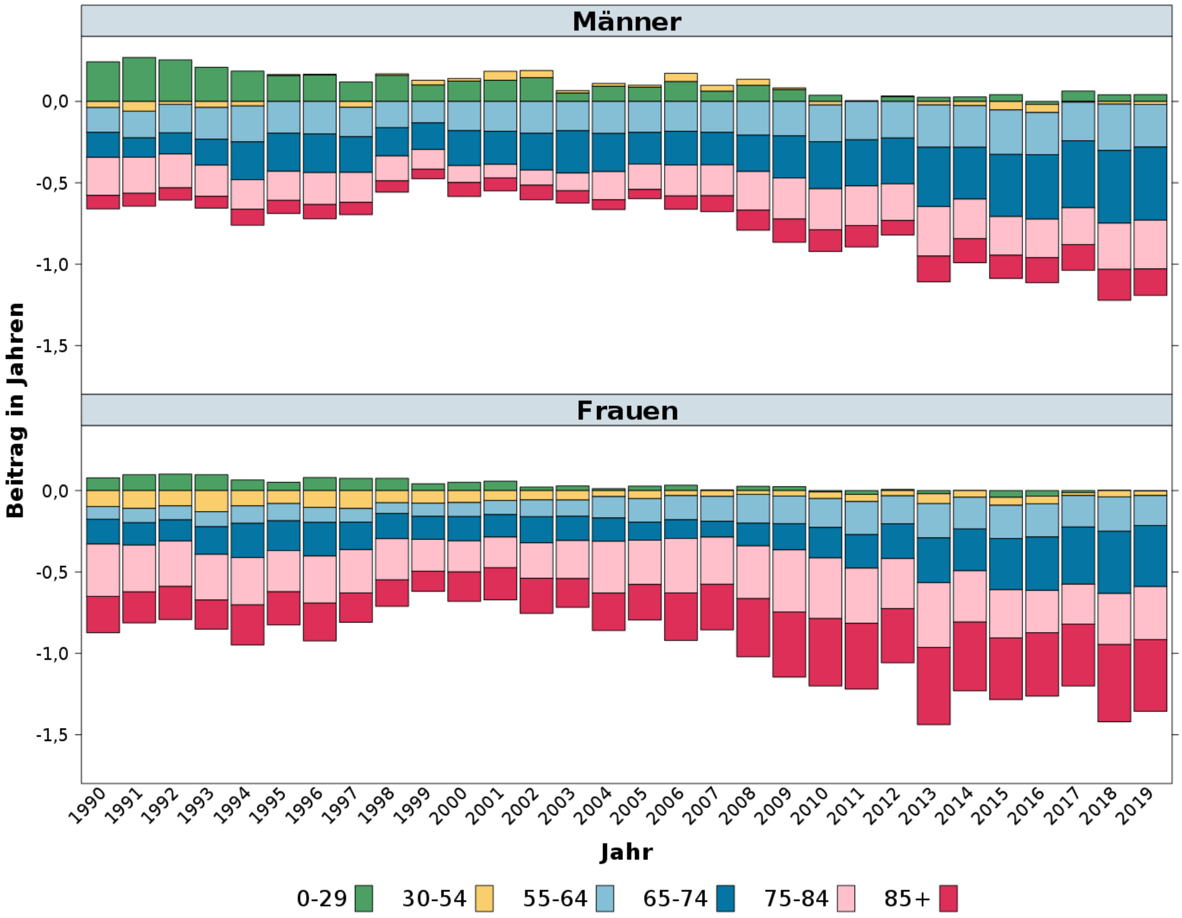

Quelle: Human Mortality Database (www.mortality.org), eigene Berechnungen und Abbildung

Abb. A2. Sterblichkeit nach großen Gruppen von Todesursachen in Deutschland und anderen westeuropäischen Ländern; beide Geschlechter, 1990 und 2017

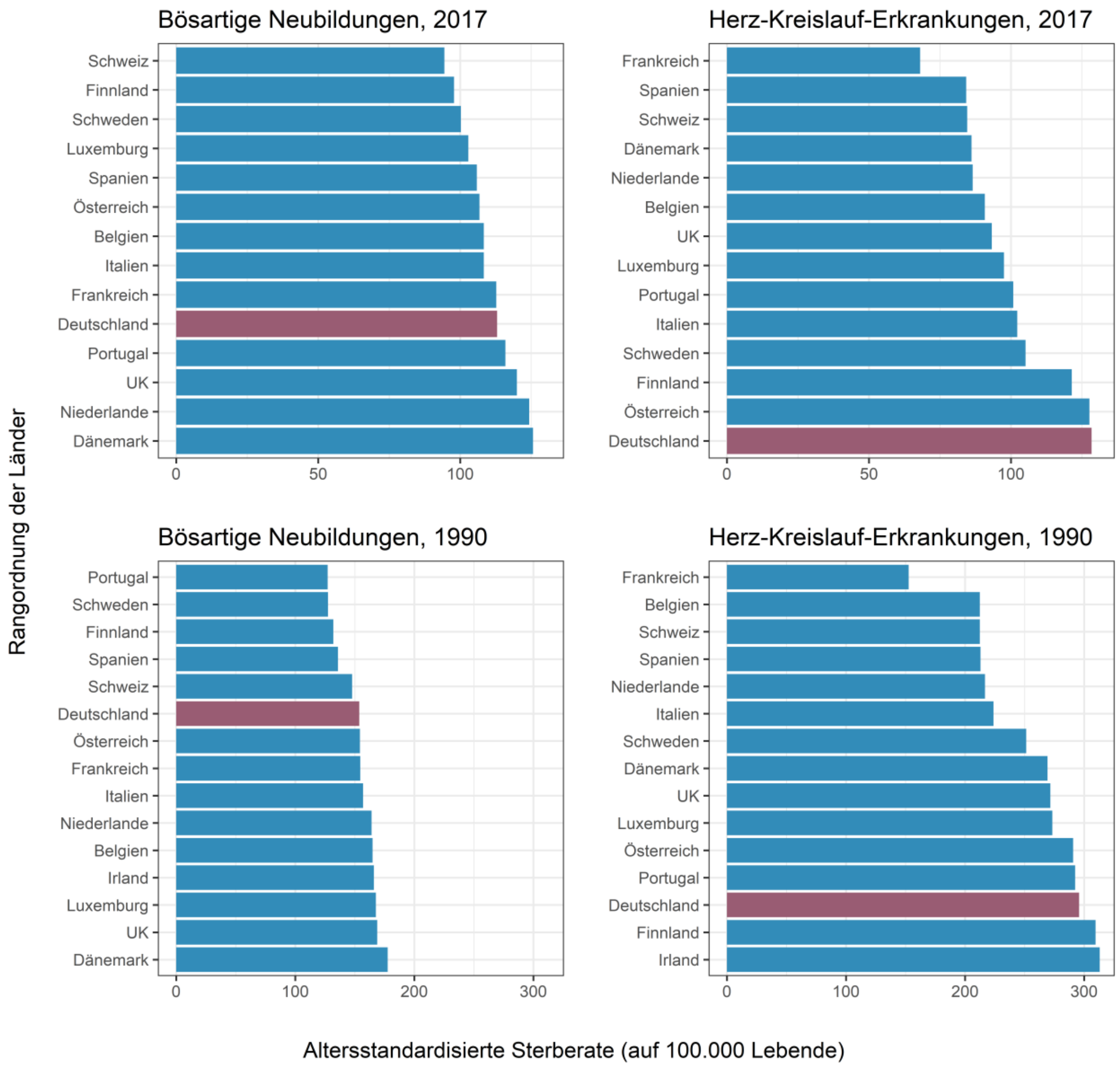

Quelle: WHO Mortality Database (https://platform.who.int/mortality), eigene Abbildung

Abb. A3. Sterblichkeit nach großen Gruppen von Todesursachen in Deutschland und anderen westeuropäischen Ländern; beide Geschlechter, 1990

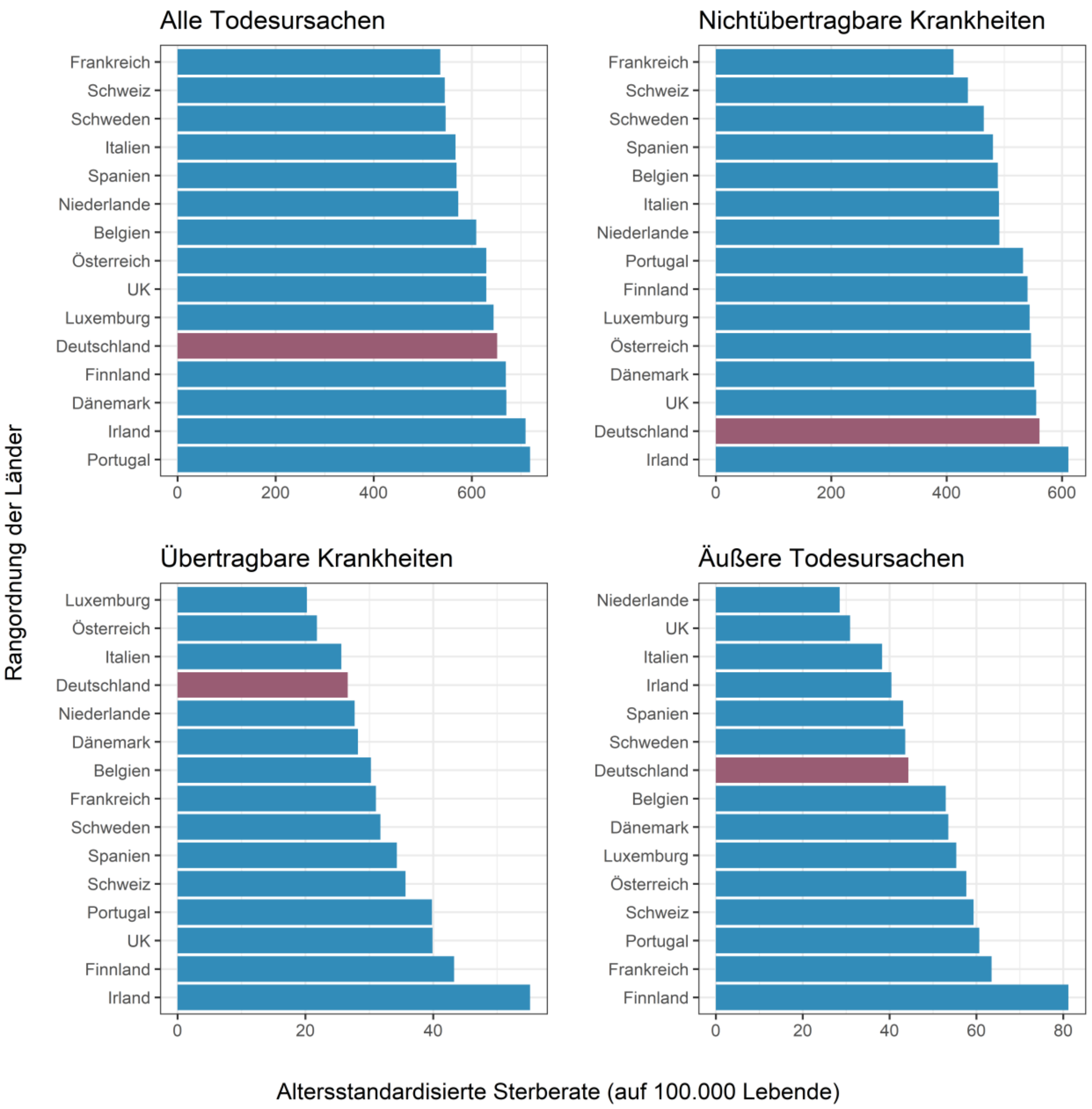

Quelle: WHO Mortality Database (https://platform.who.int/mortality), eigene Abbildung

Abb. A4. Sterblichkeit nach großen Gruppen von Todesursachen in Deutschland und anderen westeuropäischen Ländern; Männer, 2017

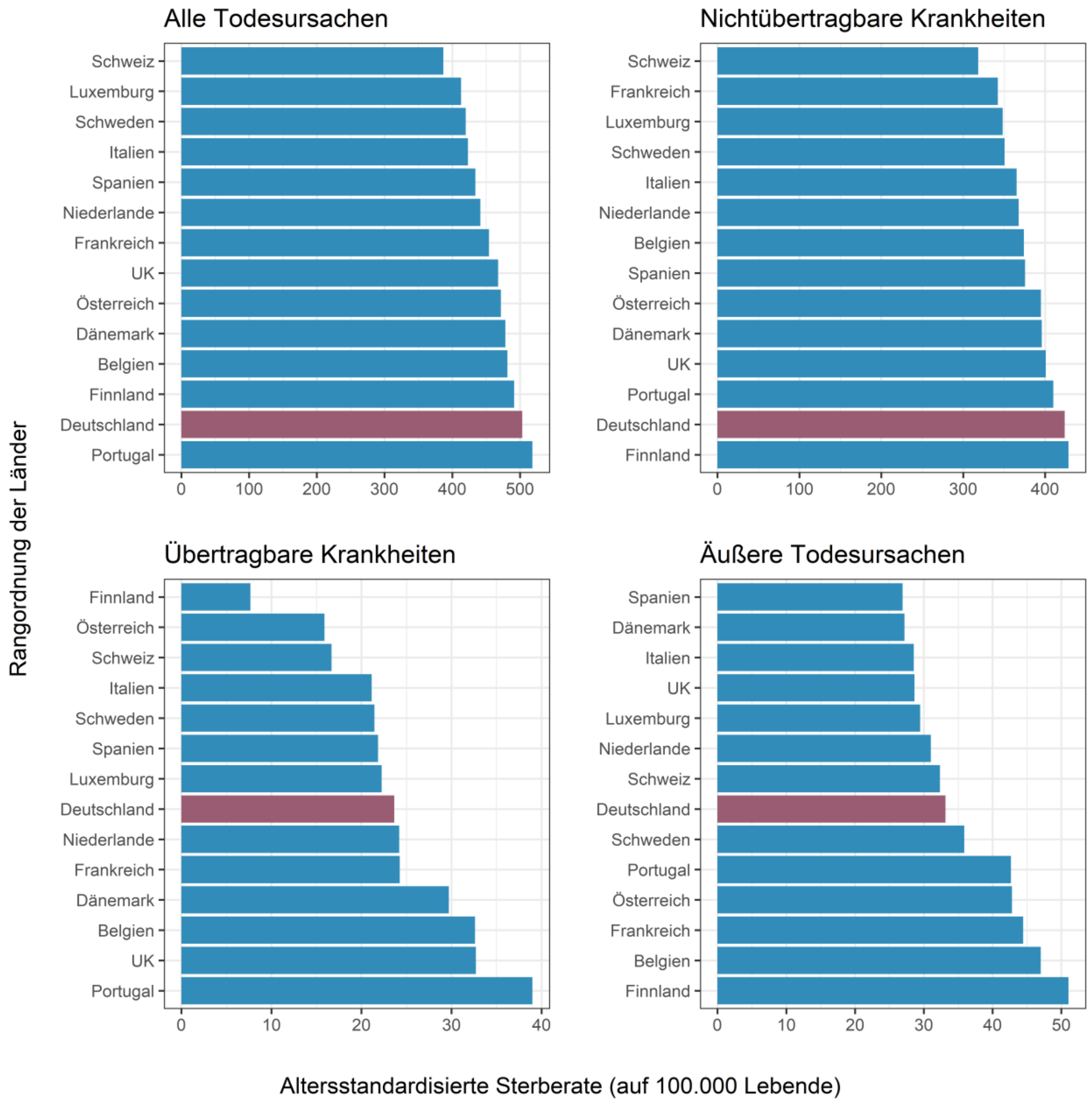

Quelle: WHO Mortality Database (https://platform.who.int/mortality), eigene Abbildung

Abb. A5. Sterblichkeit nach großen Gruppen von Todesursachen in Deutschland und anderen westeuropäischen Ländern; Frauen, 2017

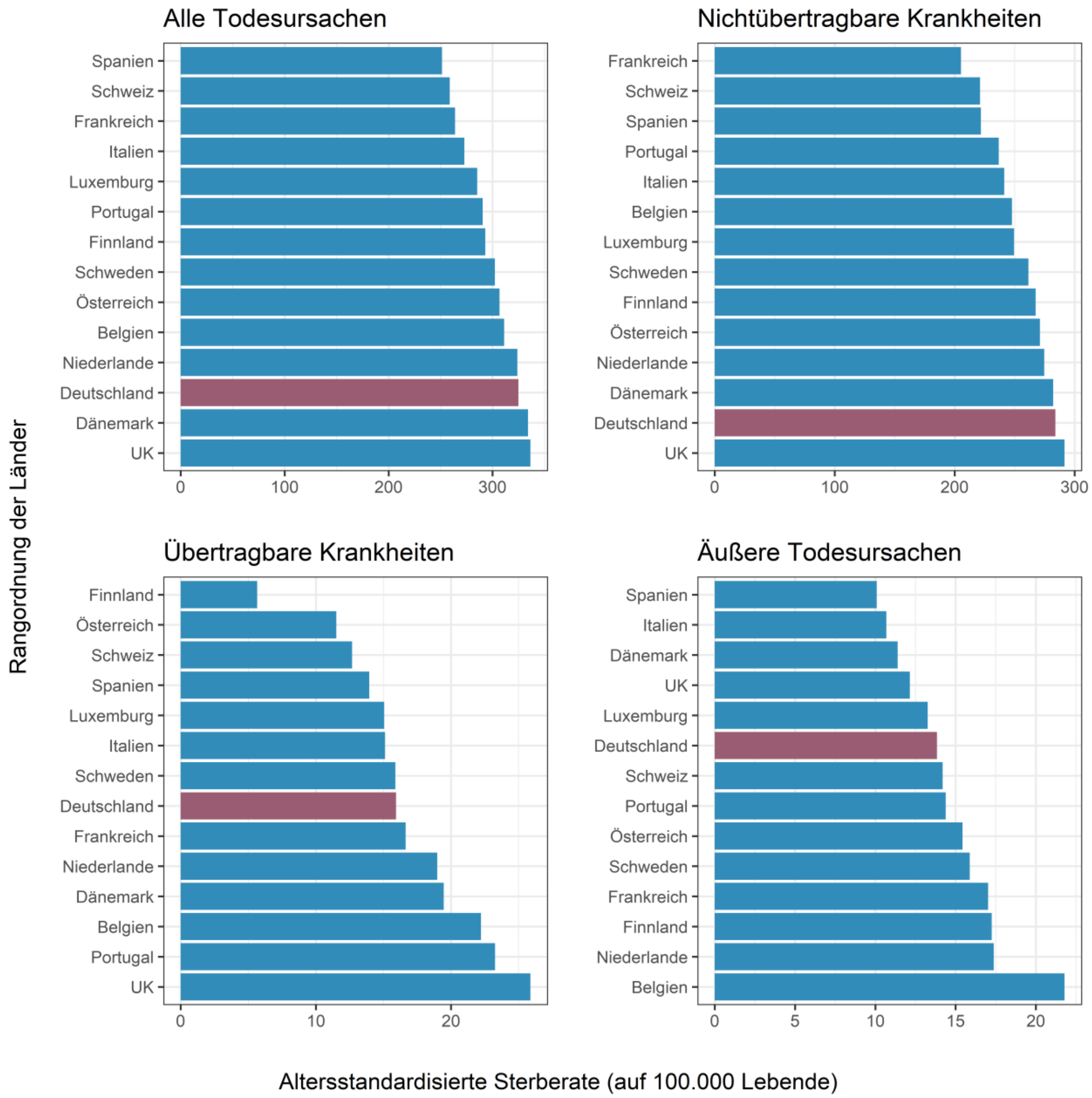

Quelle: WHO Mortality Database (https://platform.who.int/mortality), eigene Abbildung
